# Arthroscopic Repair for Posterior Shoulder Instability: Case series and surgical outcome

**DOI:** 10.1016/j.ijscr.2020.07.061

**Published:** 2020-07-31

**Authors:** Joseph Maalouly, Dany Aouad, Mohammad Darwish, Elias Saidy, Hicham Abdelnour, Robert Hanna, Georges El Rassi

**Affiliations:** St Georges University Medical Center, Beirut, Achrafieh, St Georges Street, Lebanon

**Keywords:** Shoulder, Posterior instability, Arthroscopy

## Abstract

**Purpose:**

This study evaluates outcomes of a consistent arthroscopic stabilization technique for recurrent posterior instability.

**Methods:**

79 shoulders with symptomatic posterior instability treated with arthroscopic repair and evaluated at a follow-up of 36 months. The mean age was 25.4 years of which 66 males and 13 females, and in 75% the dominant shoulder was affected. A known traumatic injury had occurred in 80%, with 25% having a documented dislocation. Arthroscopic repair was performed through an anterosuperior 12 O’clock viewing portal. Suture anchor repairs were performed in 60 cases and plication to the intact labrum in 19. A shoulder immobilizer was used for 4 weeks, followed by progressive range of motion, weightlifting at 3 months and return to contact sports at 6 months. The 79 cases had complete pre-op and post-op shoulder outcome scores.

**Results:**

Significant improvement from preoperatively to final follow-up was seen for ASES scores, from 58 to 93. All patients returned to their previous level of athletic activity. Two patients reported postoperative instability; none required reoperation. There were no other postoperative complications.

**Conclusions:**

This study represents a consecutive series of patients with recurrent posterior instability undergoing arthroscopic posterior stabilization. In this population arthroscopic posterior labral repair and capsular plication provided significant clinical improvement with low recurrence and revision rate.

## Highlights

• This study evaluates outcomes of a consistent arthroscopic stabilization technique for recurrent posterior instability.
• 79 shoulders with symptomatic posterior instability treated with arthroscopic repair and evaluated at a follow-up of 36 months.
• Arthroscopic posterior labral repair and capsular plication provided significant clinical improvement with low recurrence and revision rate.

## 1 Introduction

Posterior glenohumeral instability is a relatively rare entity, representing about 2% to 10% of all cases of instability [1]. Posterior instability can be stratified as acute traumatic event, as atraumatic instability, or as the outcome of repetitive microtrauma, in association with generalized ligamentous laxity [2,3].

Most patients with symptomatic posterior instability do not recall a precise traumatic event, like a dislocation that required reduction. It is more common for subluxation to be reported during an athletic event. Patients often have shoulder pain with activity with vague symptoms that are not localized posteriorly. Upon thorough history taking, there is usually a loss of performance and confidence in the shoulder, with a feeling of instability. Recurrent dislocation that necessitates reduction is quite rare with posterior recurrent instability while as, in anterior dislocation of the shoulder it is common [4,5].

Cases of recurrent instability may be caused by repetitive microtrauma, such as swimming, and overhead swinging in volleyball. Also, a small percentage of cases have significant ligamentous laxity [2]. Patients with both traumatic and atraumatic causes are usually competitive athletes with an age range between 15 and 40 years. The presenting symptoms are vague sometimes however, they often include an inability to participate in their usual sporting activity, pain, and multiple subluxation events [5]. In the past, the treatment for posterior glenohumeral instability consisted of physiotherapy. Surgical stabilization was reserved for patients that failed non-operative strategies. Initial reports of open repair for posterior instability showed high rates of recurrence, delay or inability to return to sports or prior activity levels, and low satisfaction of patients [[6], [7], [8], [9], [10]].

Advances in technology led to the establishment of arthroscopic stabilization as the standard of operative treatment for posterior instability. The results have been mixed in the scarce published series examining the outcomes [[11], [12], [13], [14], [15], [16]].

To this date, few studies have evaluated the results of arthroscopic posterior stabilization in athletic population with recurrent posterior instability. The purpose of this study was to evaluate outcomes of a consistent arthroscopic stabilization technique, using two portals, from a single surgeon for recurrent posterior instability. We hypothesized that arthroscopic stabilization for recurrent posterior instability in this population would provide reliable results with improvement in shoulder outcome scores and return to the preferred athletic and recreational activity levels.

## 2 Methods

### 2.1 Patient Selection

After receiving approval from the Institutional Review Board, a retrospective review of cases from 2009 to 2017 were reviewed and identified a total of 120 patients who underwent arthroscopic stabilization by the senior surgeon. After review, 79 patients were found to have undergone isolated posterior stabilization. Exclusion criteria included multidirectional instability, prior surgery, history of a seizure or neuromuscular disorder, or absence of preoperative shoulder outcome scores. 79 had completed preoperative shoulder outcome worksheets, including the American Shoulder and Elbow Surgeons (ASES) score.

The diagnosis was confirmed by thorough clinical examination and magnetic resonance (MR) imaging or MR arthrography. On clinical office examination, all patients manifested symptomatic posterior instability, with an abnormal posterior translation to and over the glenoid rim with a reproduction of their feeling of instability, apprehension, and discomfort. All had a positive posterior load-shift test.

Of 79 patients, 55 could posteriorly subluxate the shoulder with horizontal adduction at chest height with slight internal rotation. This would reduce with horizontal extension of the arm at chest height. No patient exhibited stigmata of ligamentous laxity. No sulcus sign or abnormal anterior translation. Routine shoulder radiographs were obtained including true anteroposterior, scapular Y, and axillary views, as well as MR imaging.

A trial of nonoperative treatment had failed in all patients, and all were suffering from recurrent posterior instability after a course of physical therapy, time, bracing as needed, and avoidance of their selected sport. To note, this research work has been reported in line with the PROCESS criteria [17].

### 2.2 Patient demographics

Patient demographics of the 79 patient are found in Table 1, as well their sports activities in Table 2. Most patients presented with loss of performance in their chosen athletic activity, recurrent subluxations, and pain. For the statistical analysis, bowling, swimming, golf and fitness were grouped under fitness group while as boxing, fighting, tennis, football, basketball, handball, and weightlifting as contact sports.

**Table 1:** Patient age, gender and affected side.

		Frequency	Percent
Sex	Female	13	16.46%
	Male	66	83.54%
Side	Right	60	75.95%
	Left	19	24.05%
Age	M (SD)	25.14 (6.99)	
	Min, Max	15,47

**Table 2:** Sports activities of studied population.

	Frequency	Percent
Boxing	26	32.91%
Fitness	18	22.78%
Football	9	11.39%
Basketball	7	8.86%
Golf	3	3.80%
Handball	3	3.80%
Weightlifting	3	3.80%
Fight	3	3.80%
Tennis	2	2.53%
Swimming	2	2.53%
Volleyball	2	2.53%
Bowling	1	1.27%
Total	79	100.00%

## 3 Results

Kolmogorov-Smirnov test was used to verify the normality of the ASES scores (Fig. 2). They were not normally distributed. Therefore, a Wilcoxon test was used.

General comparison between ASES scores before and after the surgery (Table 3):

**Table 3:** General comparison between ASES scores before and after the surgery.

	Mean		*Z*	*P*
	Before	After		
ASES scores	58.94	93.42	−7.724	0.000**

** Significant at level 0.01.

Wilcoxon test replaces the paired sample t-test as alternative test to study the difference between ASES scores before and after the surgery.

Based on the results in the above table, the improvement was significant the difference between the two scores is 54.47% of the score before the surgery. *p* *<* *.01*

**Fig. 1:**
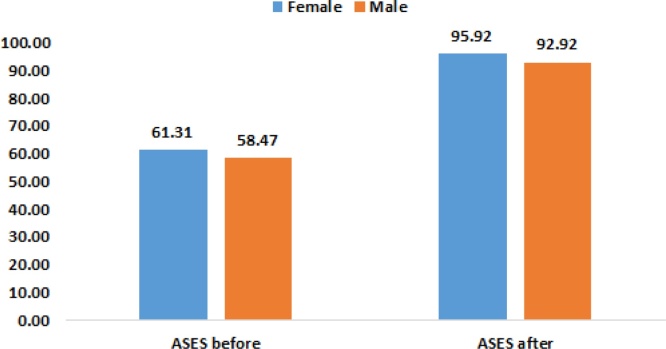
ASES score before and after surgery with reference to gender.

Gender was found to have an effect on ASES score after the surgery, *p* *<* *.05* as seen in Table 4 and Fig. 1.

**Table 4:** Gender effect on ASES scores.

	Mean Rank		Mean		*Mann-Whitney U*	*Z*	*P*
	Female	Male	Female	Male			
ASES before	48.69	38.29	61.31	58.47	316.00	−1.497	0.134
ASES after	53.58	37.33	**95.92**	**92.92**	252.50	−2.344	0.019*

**Significant at level 0.05.

**Fig. 2:**
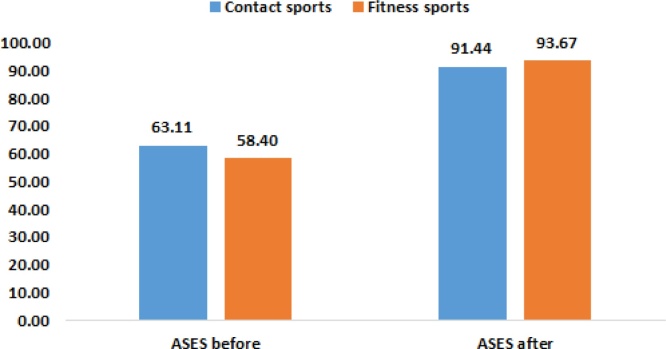
ASES score before and after surgery with reference to sports type.

Kruskal-Wallis H test replaces ANOVA as alternative test to study the effect of Lesion type on ASES scores.

The effect the type of Activity had on ASES scores is shown in Table 5.

**Table 5:** Type of activity had an effect on ASES score before the surgery, *p < *.05.

	Mean Rank		Mean		*Mann-Whitney U*	*Z*	*P*
	Contact sports	Fitness sports	Contact sports	Fitness sports			
ASES before	56.28	37.91	**63.11**	**58.40**	168.50	−2.265	0.023*
ASES after	30.89	41.17	91.44	93.67	233.00	−1.271	0.204

**Significant at level 0.05.

These results show significant improvement in ASES score postop in comparison to preop. Also, ASES score improvement was found in relation to gender and activity type. In males the improvement is likely due to reduced pain perception, larger muscles. While as in the fitness sports group, better overall scores were found because the initial damage caused the shoulder is less, the reported dislocations and subluxations were lower, and the demand postop were fewer.

## 4 Surgical Technique

All patients were placed in the beach chair position. An examination under anesthesia is performed to evaluate glenohumeral laxity and humeral translation before placing traction. This confirms the posterior instability direction in all patients. In no patient did examination lead to locked posterior dislocation. The arm was placed in shoulder traction apparatus with weights. Accessory posterior portal is used, 1 cm lateral and 1 cm inferior to the standard posterior portal, and standard anterior portals are only used. The anterior portal is made just lateral to the coracoid tip with a spinal needle localization under vision and brought into the glenohumeral joint just over the superior border of the subscapularis tendon and ensuring it reaches the posterior labrum and tangent to the glenoid. The use of two posterior portals is avoided because it damages the capsule and makes it rigid during the repair. Instruments and elevators from either the anterior or accessory posterior portal are used to elevate and prepare the labral tear, glenoid neck, and glenohumeral capsule. The working and viewing portals are interchangeable. Curved dissectors are used, rasps and curved shavers to prepare the posterior glenoid, posterior labrum tear which is completed if needed by debridement. Double loaded anchor (3 mm, Arthrex) is passed through the posterior approach without a cannula to facilitate the proper placement during anchor insertion. Suture anchors are placed on the articular margin by localizing the proper angle with a spinal needle. The posterior-inferior glenoid rim is accessed by spinal needle localization for percutaneous anchor placement. The first anchor is placed at 5 o’clock position. Then, the drill guide is percutaneously placed on the articular margin of the posteroinferior glenoid. The labrum is repaired to the glenoid with absorbable suture anchors double loaded (3 mm, Arthrex), first suture is for the inferior capsular shift, and the second for the lateral capsular shift. Usually, a small amount of capsule is taken with the suture-passing device which is then directed under the labral detachment. A PDS suture tape is passed and used to shuttle the suture anchor through the capsule and labrum for capsular shift and plication. One suture is taken from lateral to the medial aspect while the other is taken from inferior to superior direction using a scorpion needle; thus, achieving a biplanar construct. Two to three anchors are used according to the size of the tear. The use of different instruments including the spectrum, scorpion needle, lasso, peak burr to match the shape of tear and to achieve the best accessibility for the stitches.

## 5 Postoperative Rehabilitation

After surgical repair, patient’s shoulders were immobilized in a sling with an abduction pillow for three weeks. Both the pathology and the operative repair were posterior, this position puts less potential stress and tension on the repair site and the capsule. The patient was allowed to use the hand, wrist, and elbow in the sling. At three weeks, the abduction pillow was removed, and a standard sling was worn for one additional week. Pendulum exercises were instituted, and active-assisted range of motion was begun with elevation limited to 120° in order to not stress the posterior repair. At six weeks, isometric exercises for internal rotation, external rotation, the three heads of the deltoid, and the scapular rotators were begun. At eight weeks, resistive band strengthening for the shoulder was begun. Weight training was allowed at three months and return to contact sports was allowed at six months if the patient had almost normal range of motion and strength (Table 6).

**Table 6:** Advantages vs Disadvantages of the arthroscopic repair of posterior shoulder instability.

Advantages	Disadvantages
Prevent soft tissue damage to deltoid and rotator cuff muscles	Difficulty inserting the anchor in anatomical position
Easier manipulation without the use of cannula	High learning curve and operator dependant
Stable capsular shift in two different locations using double loaded anchor	Use of multiple stitches in the weak posterior capsule may lead to failure of the capsule
Prevention of further posterior labral damage	
Early recovery and rehabilitation due to minimal soft tissue damage	

## 6 Discussion

This study consisted of a consecutive series of athletic patients with isolated posterior glenohumeral instability repaired arthroscopically with a double loaded anchor by a single surgeon. No postoperative instability was reported, and no patients required reoperation for stabilization during the follow up period. Shoulder outcome methods were significantly improved from preoperative measurements, and all patients returned to their preoperative level of competitive activity. Arthroscopic instability repairs can be achieved in the beach-chair or lateral decubitus position. We opted for the beach chair position with traction, we can achieve excellent access to the lesion with preparation and repair through the anterior and posterior portals. Visualization of the lesion is essential for proper repair and restoration of anatomy. A standard anterior viewing portal is used with patients in the beach chair position for all instability repairs, both anterior and posterior portals used, are interchangeable, throughout the surgery. Some surgeons use two posterior portals in order to have good access for anchor placement. While as we opted for a single posterior portal use, to prevent further damage to the posterior capsule. For the purpose of this study, the surgical technique and postoperative rehabilitation were consistent among all patients. The described technique provides excellent visualization for anchor placement on the posterior articular margin. The placement of the double loaded anchors percutaneous allows the surgeon to place them inferiorly on the glenoid in the posterior-inferior articular margin. We believe that inferior and lateral capsular shift is essential in posterior instability because the labrum is smaller anteriorly and the capsule is thinner anteriorly.

These results are consistent with three case series. Provencher et al. described 33 consecutive patients who have undergone arthroscopic posterior stabilization with either capsular plication or a suture anchor technique [16]. Seven failed cases were reported (21%), among which four cases due to instability and three cases due to pain. Most patients reported good to excellent outcomes as measured by ASES and Western Ontario Shoulder Instability Index outcome scores. Patients with prior shoulder surgery or voluntary instability showed significantly worse outcomes. No comparison to preoperative shoulder outcome measures was made, however. Similarly, Kim et al. [11] reported 27 patients with unilateral traumatic posterior subluxation who underwent stabilization arthroscopically with labral repair and capsular plication. At a mean of 39 months’ follow-up, all patients showed significant improvement in ASES, and University of California scores. Lenart et al. [5] provided data about thirty-four consecutive shoulders with symptomatic recurrent posterior instability that were treated with arthroscopic repair using suture anchors and plication. At 36 months mean follow-up, significant improvement in ASES score from preop to postop. This study is mostly composed of a unique population of athletic patients presenting with posterior glenohumeral instability with recurrent posterior subluxations. As portrayed in Table 3, the difference in terms of ASES Score before and after surgery (58.94 vs 93.42) was statistically significant which is suggestive of reported improvement in pain and ability to perform daily living activities post-arthroscopic repair of posterior shoulder instability.

Moreover, preop and postop mean ASES scores were statistically different between males and females in which females had a higher ASES scores pre- and post-operatively. Females had a higher mean ASES Score postoperatively than males which may be related to females having a higher ASES Score preoperatively. The reported ASES Scores in the study are, to a certain extent, in contrast to the literature in which population-based studies have been consistently reporting a higher prevalence of pain in women compared to men [18,19]. The lower ASES Score in males may also be related to the initial sustained trauma where physical contact may be more intense such as in boxing or football. Moreover, postoperative ASES scores may also be influenced by gender roles in society in regard to resuming activities after surgeries and expectations. Statistically significant difference was also found between ASES Scores in terms of type of sports. Preoperatively, lower ASES Scores were reported among the fitness sport types (swimming, tennis…). This may be related to the cumulative effect of repetitive microtrauma sustained to the glenohumeral joint leading to a decrease in the ability to perform activities. The higher ASES Scores in the contact sports group can also be explained by the psycho-social aspect in which patients may not report their real level of pain due to pressure or expectations of fellow teammates or coaches. Moreover, patients in contact sport may develop a higher tolerance to pain due to the repeated intense physical contact. Postoperatively, lower ASES Scores were reported among the contact sport group. These scores may be explained by slight decrease in functionality postoperatively due to the type of trauma sustained during the sport event since contact sports are associated with more severe trauma. Moreover, these scores may be related to the patient’s loss of trust in their glenohumeral joint.

## 7 Conclusion

This study represents a series of athletic patients with recurrent posterior instability who went through two portals arthroscopic posterior stabilization with double loaded anchors. Posterior Bankart lesion should be considered for all athletes with posterior shoulder pain and pain with active anterior elevation. Few studies in the literature reported the clinical results of double loaded anchor repair of posterior Bankart lesion. The ASES Score was used to assess the patients preoperatively and postoperatively in order to assess the subjective feeling of the patient regarding pain and ability to perform activities of daily living. Thus, taking into account the patient’s satisfaction during the course of management in this population, arthroscopic posterior labral repair and capsular plication provided significant clinical improvement and low rates of recurrent instability and revision surgery. Multiple factors may be involved in the post-operative course in terms of pain and functionality with patients themselves being an important factor.

## Funding

No funds were received in support of this study.

## Ethical Approval

Ethics committee has given approval for publication.

## Consent

Written informed consent was obtained from the patient for publication of this article and accompanying images. A copy of the written consent is available for review by the Editor-in-Chief of this journal on request.

No identity identifiers are present whatsoever in the manuscript.

## Author contribution

Joseph Maalouly: contributed to the writing and editing of this article.

Dany Aouad: contributed to the writing of this article and the submission process.

Elias Saidy: contributed to the writing and referencing of this article.

Hicham Abdelnour: contributed to the writing and reviewing of this article.

Mohammad Darwish: contributed to the writing and editing of this article.

Robert Hanna: Review of literature and editing.

Georges El Rassi: Surgeon who performed the procedure, writing and editing of the article.

## Registration of Research Studies

researchregistry5668.

## Guarantor

Dr Georges El Rassi.

## Provenance and peer review

Not commissioned, externally peer-reviewed.

## Declaration of Competing Interest

The authors declare no conflict of interest regarding the publication of this article.
